# Increased Constitutive Interferon‐β Levels and Altered CD4 T Cell Homeostasis Induced by Expression of a Viral Glycoprotein

**DOI:** 10.1002/eji.70188

**Published:** 2026-04-13

**Authors:** Hanspeter Pircher, Oliver S. Thomas, Anne S. Haefke, Annette Oxenius, Thomas Boehm

**Affiliations:** ^1^ Max Planck Institute of Immunobiology and Epigenetics Freiburg Germany; ^2^ Institute of Microbiology ETH Zurich Zurich Switzerland; ^3^ Institute for Immunodeficiency Center for Chronic Immunodeficiency University Medical Center Freiburg Germany; ^4^ Max Planck Institute for Biology Tübingen Tübingen Germany

**Keywords:** homeostasis, interferon, lymphocytic choriomeningitis virus (LCMV)

## Abstract

Besides the strong Type I interferon (IFN‐I) response induced by infections, IFN‐I is also produced constitutively at lower levels. Constitutive IFN‐I production is regulated by resident microbiota and is essential for induction of efficient immune responses. Here, we demonstrate in transgenic (tg) mice that expression of a single viral envelope gene, the glycoprotein (GP) of lymphocytic choriomeningitis virus (LCMV), increased constitutive IFN‐β levels and induced numerous IFN‐stimulated genes (ISGs). In addition, self‐antigen‐driven CD44^high^CD62L^low^ memory‐phenotype CD4 T cells and regulatory CD4 T cell populations were enlarged in these LCMV GP‐tg mice; by contrast, the fraction of bona fide antigen‐specific memory CD4 T cells remained unchanged. Our study demonstrates that expression of a single viral envelope gene increased constitutive IFN‐β levels, induced upregulation of ISGs, and altered CD4 T cell homeostasis. Our results further suggest that a hitherto undefined signaling pathway capable of inducing IFN‐β expression can detect LCMV GP at the protein or transcript level.

## Graphical abstract

Constitutive interferon (IFN) production is regulated by microbiota and essential for optimal immune responses. Here, we demonstrate that expression of the glycoprotein of LCM virus upregulates constitutive IFN‐β levels and IFN‐stimulated genes (ISGs) in transgenic mice. Moreover, it increased self‐antigen‐driven memory‐phenotype CD4 T cells and effector regulatory CD4 T cells.

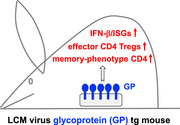

**Abbreviations**
DC: dendritic cells
EM: effector‐memory
ERV: endogenous retrovirus
GP: glycoprotein
IFNAR: interferon α/β receptor
IFN‐I: Type I interferon
ISG: IFN‐stimulated gene
LCMV: lymphocytic choriomeningitis virus
mAb: monoclonal antibody
MFI: mean fluorescence intensity
MP: memory‐phenotype
Oas: oligoadenylate synthetase
pDC: plasmacytoid dendritic cells
qRT‐PCR: quantitative reverse transcription polymerase chain reaction
Sca‐1: stem cell antigen‐1
tg: transgenic
TLR: toll‐like receptor
UPR: unfolded protein response
wt: wild type

## 1 Introduction

IFN‐β belongs to the Type I interferons (IFN‐I), a group of inflammatory cytokines that are induced in the early innate immune response against infections with viruses or other microbes. The induction of IFN‐I is triggered both by membrane‐bound toll‐like receptors (TLRs) and cytosolic molecules, including RIG‐I‐like receptors and DNA sensors such as cyclic GAMP synthase. Secreted IFN‐I bind to a heterodimeric receptor complex composed of IFNAR1 and IFNAR2 to induce expression of IFN‐stimulated genes (ISGs) in an autocrine or paracrine manner. ISGs work alone or together to mediate immune defense, to regulate cell proliferation, and to stimulate adaptive immunity [1]. Besides the strong IFN‐I response induced by acute infections, IFN‐I is also produced homeostatically, but at significantly lower levels [2, 3, 4]. Using germ‐free mice, several groups demonstrated that the gut microbiota controls these homeostatic IFN‐I expression levels [5, 6, 7, 8]. Microbiota‐regulated basal IFN‐I levels provide tonic immune stimulation that is required for optimal innate and adaptive immune responses [5, 6, 7, 9]. By contrast, constitutive IFN‐I/III expression by epithelial cells in the thymus does not require signaling through TLRs or other nucleic acid sensing receptors but is largely AIRE‐dependent [10, 11, 12]. This sterile type of IFN‐I/III production can tailor T cell selection in the thymus [10].

Infection of mice with lymphocytic choriomeningitis virus (LCMV) induces a strong IFN‐I response triggered by sensing of viral nucleic acids through TLR7/TLR9 and cytosolic MDA5/RIG‐I receptors [13, 14, 15]. Here, we surprisingly find that transgenic (tg) expression of the LCMV glycoprotein (GP) alone enhances constitutive IFN‐β levels and induces numerous ISGs. Moreover, these tg mice show an increased population of CD44^high^CD62L^low^ memory‐phenotype (MP) CD4 T cells and of regulatory CD4 T cells with an effector phenotype. Both of these CD4 T cell subsets have a high homeostatic proliferation rate and are specific for self‐antigens [16, 17, 18, 19]. The present study shows that the increased constitutive IFN‐β levels in LCMV GP‐tg mice alter the homeostasis of these self‐antigen‐driven CD4 T cells.

## 2 Results

### 2.1 LCMV GP Expression Increases Stem Cell Antigen‐1 (Sca‐1) Levels

**FIGURE 1:**
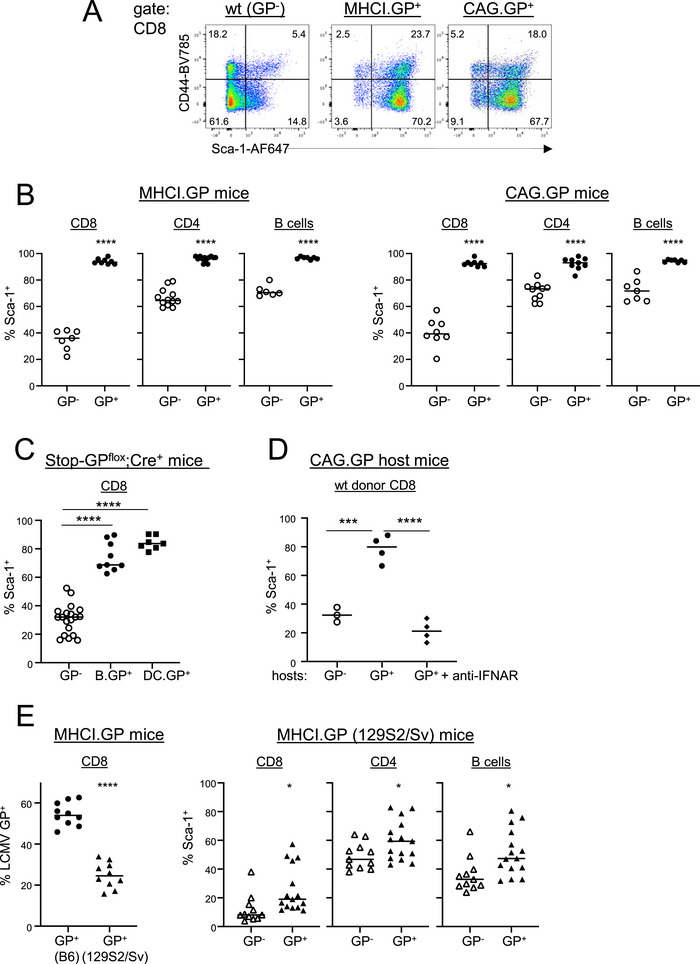
Increased Sca‐1 expression in LCMV GP‐tg mice. (A) Representative flow cytometry dot plots gated on splenic CD8 T cells from mice with the indicated genotypes. (B) Percentages of Sca‐1^+^ cells of the indicated splenic cell subsets from GP^+^ (filled circles) and GP^−^ littermate mice (open circles). (C) Percentages of Sca‐1^+^ cells of splenic CD8 T cells in mice expressing LCMV GP in B cells (B.GP^+^) or in DCs (DC.GP^+^). As control, values from GP^−^ Stop‐GP^flox^ mice are shown. (D) Percentages of Sca‐1^+^ cells of wt donor CD8 T cells after adoptive transfer into host mice with the indicated genotypes. (E) Left: LCMV GP expression in splenic CD8 T cells from MHCI.GP^+^ (B6) (filled circles) and MHCI.GP^+^ (129S2/Sv) (filled triangles) mice. Right: percentages of Sca‐1^+^ cells of the indicated splenic cell subsets from MHCI.GP^+^ (129S2/Sv) (filled triangles) and GP^−^ littermate mice (open triangles). Values from individual mice are displayed. Data are pooled from 2 to 6 independent experiments for each group (*n* = 3‐10 per group). Statistical tests: *p < 0.05; ***p < 0.001; ****p < 0.0001; unpaired *t*‐test. GP, glycoprotein; IFNAR, interferon α/β receptor; LCMV, lymphocytic choriomeningitis virus; wt, wild type.

We studied two lines of tg mice that express the GP of LCMV driven either by an MHC‐I promotor (MHCI.GP mice) or by a chicken β‐actin promotor (CAG.GP mice). Intriguingly, splenic CD8 T cells from both tg lines, but not from wild type (wt) mice, expressed high levels of the Sca‐1 marker (Figure 1A), although only minimal expression of LCMV GP is observed in CD8 T cells of the CAG.GP tg line (Figure S1B). The Sca‐1 (Ly6A/E) is a well‐known marker for hematopoietic cells, and it is induced by IFN‐I [20, 21, 22]. As in CD8 T cells, Sca‐1 was also upregulated in CD4 T cells and in B cells from both GP^+^ tg lines (Figure 1B), again in discordance with tg LCMV GP expression profiles (Figure S1).

Given these observations, we determined whether upregulation of Sca‐1 by LCMV GP was the result of a cell extrinsic or intrinsic process. To this end, we analyzed mice expressing LCMV GP specifically in B cells or in dendritic cells (DCs). These mice were generated by mating Stop‐GP^flox^ mice [23] with mice expressing cre recombinase in B cells (mb1‐cre [24]) or in DCs (CD11c‐cre [25]). Remarkably, B cell‐ or DC‐specific LCMV GP expression was capable of inducing Sca‐1 upregulation in CD8 T cells, clearly demonstrating that LCMV GP expression induces Sca‐1 upregulation in a cell extrinsic manner (Figure 1C).

As Sca‐1 expression is induced by IFN‐I [20, 21], we hypothesized that LCMV GP expression led to increased systemic IFN‐I levels. To provide further evidence for this hypothesis, we adoptively transferred CD8 T cells from wt B6.Thy1.1^+^ mice into CAG.GP^+^ mice. After 5 days, Sca‐1 expression in the transferred CD8 T cell population was determined. wt donor CD8 T cells upregulated Sca‐1 expression in GP^+^ but not in GP^−^ host mice, and anti‐interferon α/β receptor (IFNAR) antibody treatment blocked this upregulation (Figure 1D). Next, we used Ly6C as an additional marker also known to be induced by IFN‐I in CD8 T cells [26, 27]. Like Sca‐1, expression of Ly6C in naïve CD44^low^CD8 T cells was markedly upregulated in MHCI.GP^+^ and CAG.GP^+^ mice (Figure S2A). After adoptive transfer into CAG.GP^+^ mice, Ly6C upregulation of wt donor CD8 T cells was also abolished by anti‐IFNAR antibodies (Figure S2B).

Unless stated otherwise, the mice described here were maintained on a C57BL/6 (B6) background. MHCI.GP^+^ mice backcrossed to the 129S2/Sv background for three generations expressed LCMV GP and Sca‐1 at considerably lower levels (Figure 1E , left). It is, however, important to state that the frequencies of Sca‐1^+^ cells in non‐tg 129S2/Sv mice were also considerably lower than in B6 mice. Thus, Sca‐1 upregulation in MHCI.GP^+^ mice seems to correlate in a positive manner with GP expression levels, but background effects independent of GP expression might contribute to this phenomenon. Taken together, these data strongly suggest that LCMV GP expression in tg mice leads to Sca‐1 upregulation by increased IFN‐I levels acting in trans.

### 2.2 LCMV GP Expression Upregulates IFN‐β and ISGs

**FIGURE 2:**
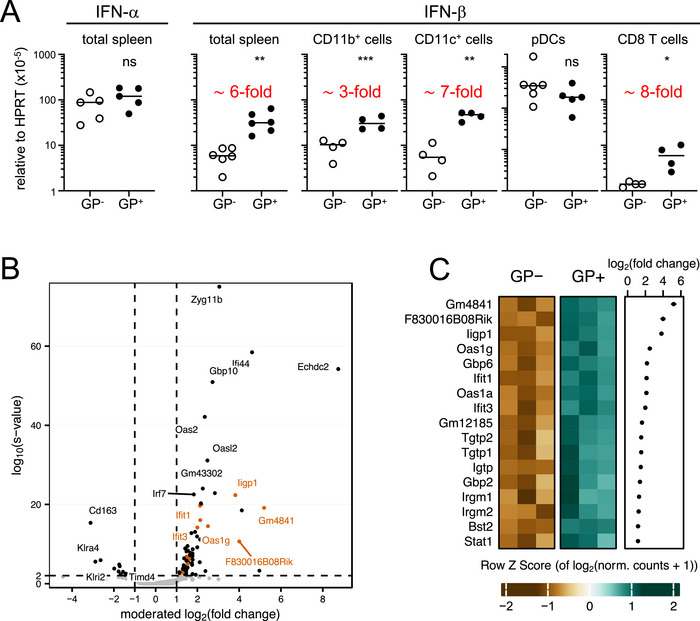
Induction of IFN‐β and of ISGs in MHCI.GP^+^ mice. (A) IFN‐I expression normalized to HPRT in total splenic tissue and in the indicated splenic cell subsets of MHCI.GP^+^ and MHCI.GP^−^ mice as determined by qRT‐PCR. Values from individual mice are displayed. Red numbers in the plots indicate approximate ratios of sample means in GP^+^ compared to GP^−^ mice. Data are pooled from 2 to 6 independent experiments for each group (*n* = 4–6 per group). Statistical tests: ns, not significant; *p < 0.05; **p < 0.01; ***p < 0.001; unpaired *t*‐test. (B and C) RNA‐seq of splenic lymphocytes from MHCI.GP^+^ and MHCI.GP^−^ mice (*n* = 3 per group). (B) Volcano plot of differential expression analysis results showing log_2_(fold change) by −log_10_(*s* value). Genes reaching significance thresholds (*s* value ≤ 0.01, testing for |log_2_(fold change)| ≥ 1; dashed lines) are black. Genes belonging to the “Response to Interferon‐beta” gene set are marked in orange. Other genes are gray. (C) Heatmap showing *Z* scores of all significant genes annotated with the GO term “Response to Interferon‐beta.” log_2_(fold change) ± standard error is indicated for each gene. GP, glycoprotein; pDC, plasmacytoid dendritic cell.

To directly demonstrate that LCMV GP upregulates IFN‐I in tg mice, IFN‐I mRNA levels were determined by quantitative reverse transcription polymerase chain reaction (qRT‐PCR). We first used RNA from total splenic tissue of MHCI.GP^+^ and age‐matched tg‐negative littermates (MHCI.GP^−^ mice). Overall, IFN‐I mRNA levels in the spleens of these noninfected mice were detectable but very low, confirming previous observations by others [7, 12]. To quantify IFN‐α mRNA levels, we used pan IFN‐α primers for qRT‐PCR but did not observe a difference between MHCI.GP^+^ and MHCI.GP^−^ mice (Figure 2A, first panel). However, total splenic tissue of MHCI.GP^+^ mice contained about ∼6‐fold increased IFN‐β mRNA levels (Figure 2A, second panel). We also determined IFN‐β mRNA levels in isolated splenic CD11b^+^ macrophages, CD11c^+^ DCs, plasmacytoid DCs (pDC), and CD8 T cells. In CD11b^+^ and CD11c^+^ cells, IFN‐β mRNA levels were increased in MHCI.GP^+^ mice about ∼3‐ and ∼7‐fold, respectively (Figure 2A, third and fourth panel). Surprisingly, IFN‐β mRNA levels did not significantly differ in pDCs of MHCI.GP^+^ and MHCI.GP^−^ mice, although their overall levels were increased compared to CD11b^+^ and CD11c^+^ cells (Figure 2A, fifth panel). In MHCI.GP^+^ mice, CD8 T cells predominantly expressed LCMV GP (Figure S1A). IFN‐β transcripts in CD8 T cells from MHCI.GP^+^ mice were upregulated, but the overall expression levels were considerably lower compared to CD11b^+^ and CD11c^+^ cells (Figure 2A, rightmost panel). In sum, these data show that LCMV GP expression in tg mice increased IFN‐β mRNA levels in splenic tissue, including CD11c^+^ and CD11b^+^ cells, but not pDCs.

To determine whether the elevated IFN‐β mRNA levels led to increased expression of ISGs, we performed RNA‐seq experiments using splenic lymphocytes from MHCI.GP^+^ and MHCI.GP^−^ mice. In lymphocytes from MHCI.GP^+^ mice, 80 genes were significantly upregulated and 13 genes were significantly downregulated with a fold change of two or more in either direction (Figure 2B). Overrepresentation analysis (ORA) further revealed an enrichment in the GO term “Response to Interferon‐beta” and other interferon‐related gene sets (Figure S3). Most of the upregulated genes belonged to families that are associated with IFN signaling. This includes IFN‐inducible GTPases [28, 29], IFN‐induced proteins with tetratricopeptide repeats (IFITs) [30], and oligoadenylate synthetases (Oas) [31]. In addition, we observed an increase of ISGs with antiviral activities such as Ifi44 [32], Ifi27l2a [33], Isg15 [34], Rsad2 [35], Parp12 [36], and Zyg11b [37] (Figure 2C and Figure S4).

Nucleic acid replication intermediates of endogenous retroviruses (ERVs) have the potential to engage multiple innate sensors [38]. As retroviral vectors can be efficiently pseudo‐typed with LCMV GP [39], we explored the possibility that LCMV GP expression could rescue defective ERVs and lead to increased ERV production. However, MHCI.GP^+^ mice did not exhibit a transcriptional increase in LINE, SINE, or LTR/ERV repeat element families (Figure S3). Similarly, MHCI.GP^+^ mice did not show an enrichment in gene transcripts involved in the unfolded protein response (UPR), arguing against the notion that the increased IFN‐β levels were due to the accumulation of misfolded LCMV GP (Figure S3). Taken together, these data demonstrate that the increased constitutive IFN‐β levels induced by LCMV GP upregulated numerous ISGs in MHCI.GP^+^ mice.

### 2.3 Preferential Expression of Upregulated ISGs in Spleen Compared to Thymus

**FIGURE 3:**
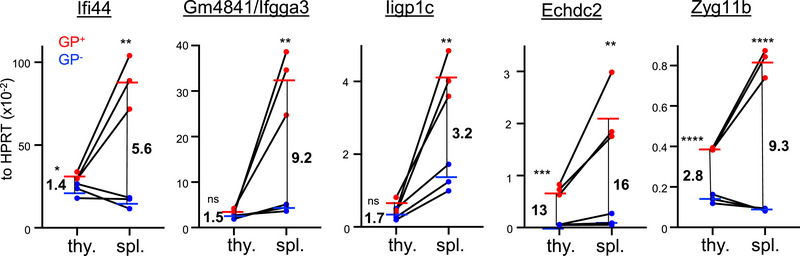
Preferential induction of ISGs in spleen compared to thymus in MHCI.GP^+^ mice. Expression of the indicated ISGs normalized to HPRT in thymus (thy) and spleen (spl) of MHCI.GP^+^ (red circles) and MHCI.GP^−^ (blue circles) mice as determined by qRT‐PCR. Values from the same individual mouse are connected by a black line. Pooled data from three independent experiments for each group (*n* = 3 per group) are shown. Numbers in the plots indicate approximate ratios of sample means in MHCI.GP^+^ compared to MHCI.GP^−^ mice. Statistical tests: *p < 0.05; **p < 0.01; ***p < 0.001; ****p < 0.0001; unpaired *t*‐test. ns, not significant.

Next, we determined the expression levels of five of the most upregulated genes (*Echdc2, Zyg11b, Gm4841, Ifi44*, and *lipgp1c*) by quantitative RT‐PCR in thymus and spleen of MHCI.GP^+^ and MHCI.GP^−^ mice (Figure 3). Confirming the RNA‐seq data, all five examined genes were strongly upregulated in spleens of MHCI.GP^+^ mice. Interestingly, expression levels of three of the five analyzed genes (*Ifi44*, *Gm4841*, and *lipgp1c*) remained essentially unchanged in the thymi of tg mice. The other two genes (*Echdc2* and *Zyg11b*) were induced in MHCI.GP^+^ thymi, but their expression levels were still considerably lower when compared to tg spleens. Hence, some of the differentially expressed genes in MHCI.GP^+^ mice bear the characteristics of peripherally induced self‐antigens.

### 2.4 LCMV GP Expression Increases Conventional and Regulatory CD4 T Cells With an Activated Phenotype

**FIGURE 4:**
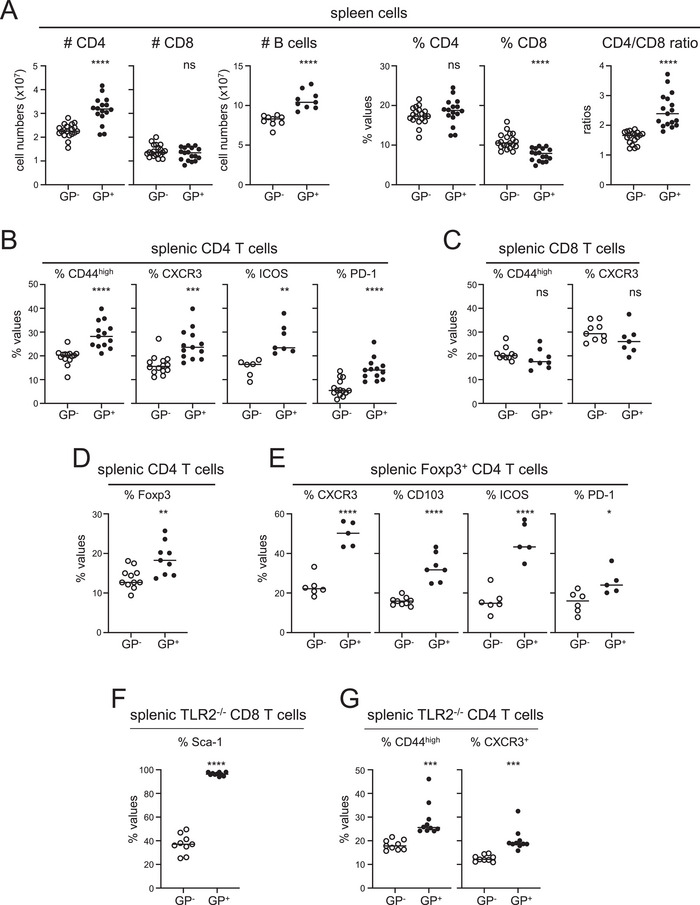
Increase of CD4 T cells with an activated phenotype in MHCI.GP^+^ mice. (A) Absolute cell numbers and percentages of the indicated cell subsets in spleen of MHCI.GP^+^ (filled circles) and MHCI.GP^−^ (open circles) mice. (B and C) Expression of the indicated markers in percent of splenic CD4 (B) and CD8 T cells (C). (D) Percentages Foxp3^+^ cells of splenic CD4 T cells. (E) Expression of the indicated markers in percent of splenic Foxp3^+^CD4 T cells. (F) Percentages of Sca‐1^+^ cells of splenic CD8 T cells from MHCI.GP^+^;TLR2^−/−^ (filled circles) and MHCI.GP^−^;TLR2^−/−^ littermates (open circles). (G) Percentages of CD44^high^ (left) and of CXCR3^+^ cells (right) of splenic CD4 T cells from MHCI.GP^+^;TLR2^−/−^ (filled circles) and MHCI.GP^−^;TLR2^−/−^ littermates (open circles). Values from individual mice are displayed. Data are pooled from 4 to 10 independent experiments for each group (*n* = 5–20 per group). Statistical tests: *p < 0.05; **p < 0.01; ***p < 0.001; ****p < 0.0001; unpaired *t*‐test. GP, glycoprotein; ns, not significant.

Next, we determined whether MHCI.GP^+^ mice exhibited additional alterations besides increased IFN‐β and ISG levels. Compared to age‐matched tg‐negative littermates, MHCI.GP^+^ mice contained increased numbers of splenic CD4 T cells and B cells by 38% and 31%, respectively (Figure 4A, left). CD8 T cell numbers were comparable to wt mice, but due to the increased CD4 and B cells, their relative abundance decreased in MHCI.GP^+^ mice. This led to an increase in CD4/CD8 ratios from ∼1.6 in MHCI.GP^−^ to ∼2.5 in MHCI.GP^+^ mice (Figure 4A, right). Flow cytometry using the activation markers CD44, CXCR3, ICOS, and PD‐1 revealed significantly increased proportions of activated CD4 T cells in the spleen of MHCI.GP^+^ mice (Figure 4B). MHCI.GP^+^ mice also displayed an increase of these activation markers within the subset of conventional Foxp3^−^CD4 T cells (Figure S5A). In contrast, the proportions of CD44^high^ and of CXCR3^+^ cells within the CD8 T cell subset were not altered (Figure 4C). Interestingly, MHCI.GP^+^ mice also contained increased percentages of Foxp3^+^ cells of total CD4 T cells by 36% on average (Figure 4D). In addition, a larger fraction of Foxp3^+^ regulatory CD4 T cells in MHCI.GP^+^ mice showed an activated effector phenotype (Figure 4E). Within the B cell subset of MHCI.GP^+^ mice, we observed an increase in IgM^−^IgD^−^ switched memory (1.04%–1.54%), Fas^+^GL7^+^ germinal center (0.12%–0.54%), and CXCR3^+^ cells (2.0%–3.96%) (Figure S5B). Furthermore, B cells from these mice showed upregulated expression levels of CD86, MHC‐I (K^b^), and MHC‐II (I‐A^b^) (Figure S5C).

Importantly, the increase in activated CD4 T cells was also seen in the independent CAG.GP^+^ tg line, supporting the notion that this effect reflected the biological consequence of LCMV GP expression. In comparison to MHCI.GP^+^ mice, the activated CD4 T cell phenotype in CAG.GP^+^ mice was somewhat less pronounced, but increases in CD4 T cells, CD4/CD8 ratios, and CD4 T cell activation markers both in total and in regulatory CD4 T cells were also observed (Figure S6).

To examine whether the increased proportion of activated CD4 T cells in MHCI.GP^+^ mice was due to a failure in tolerance induction resulting in LCMV GP‐induced activation, the numbers and the CD44 phenotype of LCMV GP‐specific CD4 T cells were determined by pMHC tetramer (tet)‐based enrichment using the immunodominant LCMV gp66 CD4 T cell epitope. However, the numbers of gp66‐tet^+^ CD4 T cells in MHCI.GP^+^ mice were not increased and even trended slightly lower (Figure S7A,B). In addition, most gp66‐tet^+^ CD4 T cells in MHCI.GP^+^ mice showed low tetramer staining intensities and a nonactivated CD44^low^ phenotype in contrast to total CD4 T cells (Figure S7C).

Zhou et al. proposed that TLR2 serves as a receptor for LCMV proteins [40], but we found that LCMV GP expression in TLR2‐deficient mice also upregulated Sca‐1 expression in CD8 T cells and increased CD4 T cell activation markers (Figure 4F,G), ruling out a role of this sensor in the model described here. In sum, these data demonstrate that LCMV GP expression in tg mice leads to a remarkable activated CD4 T cell phenotype.

### 2.5 Decrease of the Activated CD4 T Cell Phenotype in the Absence of IFN‐I Signaling

**FIGURE 5:**
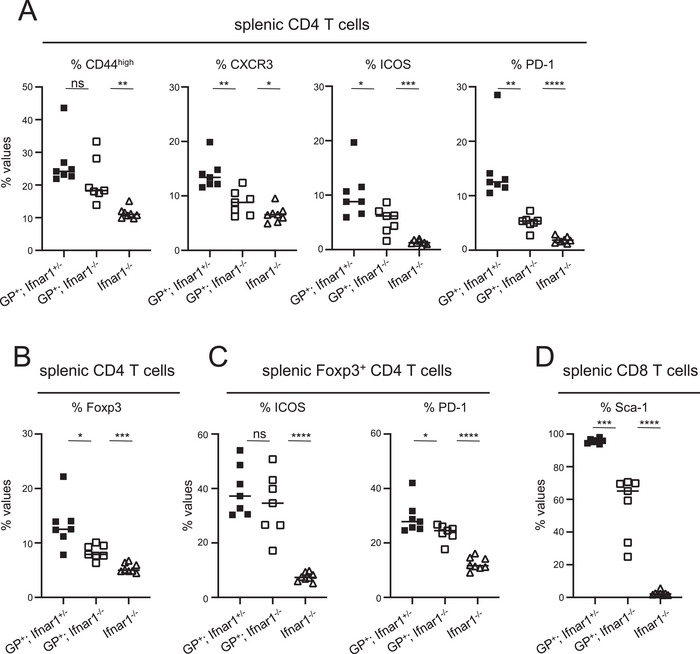
Decrease of the activated CD4 T cell phenotype in IFN‐I signaling‐deficient MHCI.GP^+^ mice. (A) Expression of the indicated markers in percent of splenic CD4 T cells from mice with the indicated genotypes. (B) Percentages Foxp3^+^ cells of splenic CD4 T cells. (C) Expression of the indicated markers in percent of splenic Foxp3^+^CD4 T cells. (D) Percentages of Sca‐1^+^ cells of splenic CD8 T cells. Values from individual mice are displayed. Data are pooled from two independent experiments for each group (*n* = 7 per group). Statistical tests: *p < 0.05; **p < 0.01; ***p < 0.001; ****p < 0.0001; unpaired *t*‐test. GP, glycoprotein; Ifnar1, IFN‐I receptor 1; ns, not significant.

To examine whether the activated CD4 T cell phenotype in MHCI.GP^+^ mice is dependent on IFN‐I signaling, we analyzed mice deficient in IFN‐I receptor 1 (Ifnar1) expression. As above, we assessed the activation phenotype in spleen using CD44, CXCR3, PD‐1, and ICOS as markers. MHCI.GP^+^;Ifnar1^−/−^ mice showed a significantly decreased CD4 T cell activation phenotype compared to IFN‐I receptor sufficient littermates expressing LCMV GP (Figure 5A). Nonetheless, the fractions of activated cells in MHCI.GP^+^;Ifnar1^−/−^ mice were still greater than those observed in Ifnar1^−/−^ littermates without LCMV GP expression (Figure 5A). Similarly, frequencies of splenic Foxp3^+^CD4 T cells with an activated phenotype were decreased in MHCI.GP^+^;Ifnar1^−/−^ mice compared to MHCI.GP^+^;Ifnar1^+/−^ mice but still remained above the values in Ifnar1^−/−^ mice without LCMV GP expression (Figure 5B,C). Moreover, although MHCI.GP^+^;Ifnar1^−/−^ mice showed considerably lower Sca‐1 levels in CD8 T cells compared to MHCI.GP^+^;Ifnar1^+/−^ mice, the values were still higher than those for Ifnar1^−/−^ littermates lacking LCMV GP expression (Figure 5D). Taken together, these results indicate that the increased IFN‐β levels in MHCI.GP^+^ mice were, at least partially, responsible for the activated CD4 T cell phenotype and for the upregulated Sca‐1 levels. The observation that these alterations were not entirely Ifnar1‐dependent further suggests that, besides IFN‐I, additional factors with activation potential are also induced by LCMV GP expression.

### 2.6 Increase of MP but Not of Bona Fide Memory CD4 T Cells

**FIGURE 6:**
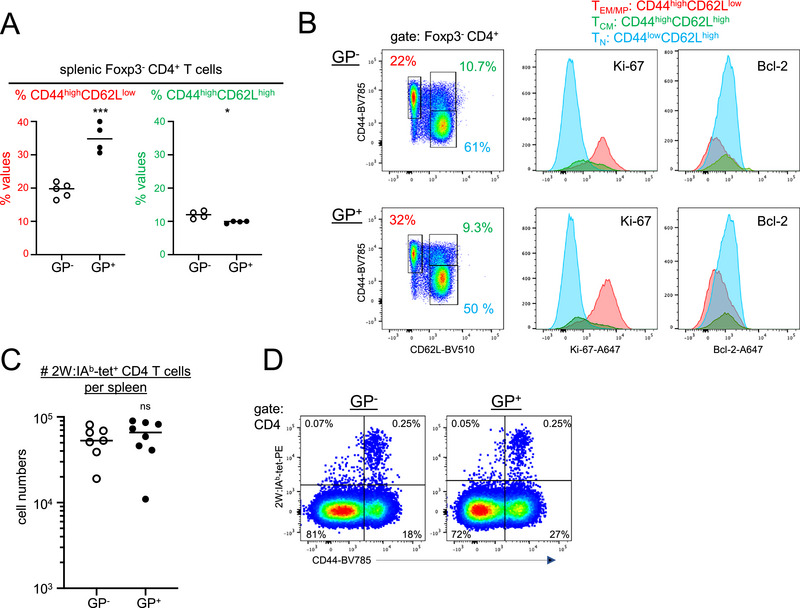
Increase of memory‐phenotype but not of antigen‐induced CD4 T cells in MHCI.GP^+^ mice. (A) Percentages of CD44^high^CD62L^low^ and CD44^high^CD62L^high^ cells of Foxp3^−^CD4 T cells from spleen of MHCI.GP^+^ (filled circles) and MHCI.GP^−^ (open circles) mice. (B) The flow cytometry histograms show expression of Ki‐67 and Bcl‐2 of naïve T_N_ (blue), T_CM_ (green), and T_EM/MP_ (red) Foxp3^−^CD4 T cells. The gates used for the individual populations in the histograms are depicted in the dot plots on the left. (C) Absolute numbers of 2W:I‐A^b^ tet^+^ CD4 T cells in spleen of MHCI.GP^+^ (filled circles) and MHCI.GP^−^ (open circles) mice immunized with the 2W peptide. (D) Representative flow cytometry dot plots gated on splenic CD4 T cells of 2W peptide‐immunized mice. Values from individual mice are displayed. Data are pooled from 2 to 4 independent experiments for each group (*n* = 4–8 per group). Statistical tests: *p < 0.05; ***p < 0.001; unpaired *t*‐test. GP, glycoprotein; ns, not significant.

Memory T cells are often partitioned into CD44^high^CD62L^high^ central memory and CD44^high^CD62L^low^ effector memory cells [41]. In addition, naïve CD4 T cells may also proliferate in response to self‐antigens to acquire a CD44^high^CD62L^low^ phenotype [19]. These MP CD4 T cells differ from foreign antigen‐specific memory T cells by a high turnover rate and low Bcl‐2 expression [16]. In MHCI.GP^+^ mice, the proportions of CD44^high^CD62L^low^ but not of central CD44^high^ CD62L^high^ memory CD4 T cells were increased (Figure 6A). These CD44^high^CD62L^low^CD4 T cells were proliferating at a high level as assessed by the Ki‐67 marker expression, and they expressed low levels of Bcl‐2, indicating that they indeed predominantly belonged to the MP CD4 T cell subset (Figure 6B). Of note, most of the CD44^high^CD62L^low^CD4 T cells in wt mice were also Ki‐67^high^Bcl‐2^low^, but their numbers were considerably lower than in MHCI.GP^+^ mice. Thus, LCMV GP expression in MHCI.GP^+^ mice specifically enlarges the MP CD4 T cell subset in addition to regulatory CD4 T cells with an effector phenotype.

To examine whether tg LCMV GP expression also increases foreign antigen‐specific effector/memory CD4 T cells, MHCI.GP mice were immunized with the 2W model peptide [42]. After 10 days, the numbers of 2W‐specific effector/memory CD4 T cells were determined by 2W:I‐A^b^ tetramer (tet) staining. The numbers of the induced 2W:I‐A^b^ tet^+^ CD4 T cells in MHCI.GP^+^ and MHCI.GP^−^ mice were comparable (Figure 6C), and 2W:I‐A^b^ tet^+^ CD4 T cells in both types of mice expressed high levels of CD44 (Figure 6D). Taken together, these data demonstrate that MHCI.GP^+^ mice exhibit increased numbers of self‐antigen‐driven MP CD4 T cells but not of foreign antigen‐induced effector/memory CD4 T cells.

### 2.7 CD44^high^ CD4 T Cells in LCMV GP Expressing Mice Have a Distinct TCR Repertoire

**FIGURE 7:**
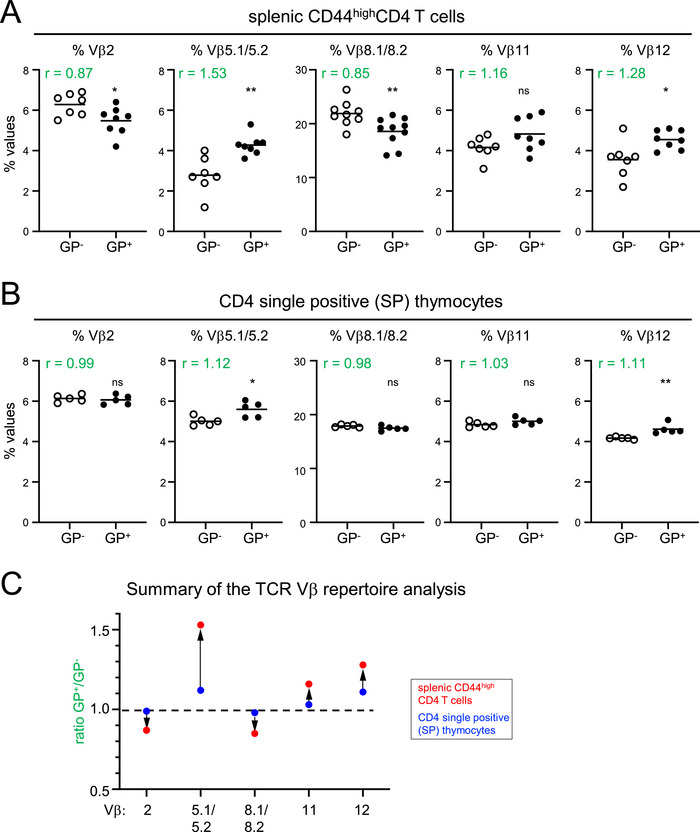
Distinct TCR Vβ repertoire of CD44^high^CD4 T cells in MHCI.GP^+^ mice. Percentages of Vβ2^+^, Vβ5.1/5.2^+^, Vβ8.1/8.2^+^, Vβ11^+^, and Vβ12^+^ cells of splenic CD44^high^CD4 T cells (A) and of CD4 single positive (SP) thymocytes (B) from MHCI.GP^+^ (filled circles) and MHCI.GP^−^ (open circles) mice. In each Vβ*_x_* plot, the ratio (*r*) of % Vβ*_x_* ^+^ cells in GP^+^ versus GP^−^ mice is indicated in green. (C) Summary of the TCR Vβ repertoire analysis in thymic (blue dots) versus splenic (red dots) CD4 T cells. Dots represent the ratio (*r*) of % Vβ*_x_* ^+^ cells in GP^+^ versus GP^−^ mice as given in the plots shown in (A and B). Values from individual mice are displayed. Data are pooled from 4 to 6 independent experiments for each group (*n* = 5–8 per group). Statistical tests: *p < 0.05; **p < 0.01; unpaired t‐test. GP, glycoprotein; ns, not significant.

Finally, we analyzed the TCR repertoire of CD44^high^CD4 T cells in MHCI.GP mice with a panel of TCR Vβ‐specific antibodies known to bind to a significant proportion of CD4 T cells. The data show that the TCR Vβ repertoire of CD44^high^CD4 T cells in MHCI.GP^+^ mice was distinct from their littermates without LCMV GP expression (Figure 7A). There was an increase in the proportions of TCR Vβ5^+^ and Vβ12^+^ cells and a decrease in Vβ2^+^ and Vβ8.1/8.2^+^ cells in MHCI.GP^+^ mice. There was also a slight, yet nonsignificant, increase in Vβ11 expression in MHCI.GP^+^ mice. To examine whether these alterations already occurred during thymic selection, the repertoire of CD4 single‐positive (SP) thymocytes was also determined using the same set of TCR Vβ‐specific antibodies. We observed the same trend with increased Vβ5^+^/Vβ12^+^ and decreased Vβ2^+^/Vβ8.1/8.2^+^ expression in the thymic CD4 SP subset, but the overall differences were much less pronounced compared to peripheral CD44^high^CD4 T cells (Figure 7B,C). Together, these results indicate that tg LCMV GP expression slightly altered thymic and, more robustly, peripheral selection of CD4 T cells.

## 3 Discussion

Here, we show that tg expression of a single viral envelope gene, the GP of LCMV, increased IFN‐β expression and induced numerous ISGs in vivo. It is well known that infection of mice with LCMV leads to a rapid and robust IFN‐I response. Sensors such as MDA5 and TLR7 that detect viral RNA in the cytosol or in endosomes of infected cells mediate this response [13, 14, 15]. In contrast, the LCMV GP transcripts in the tg mice studied here are derived from a transgene that is integrated into the mouse genome. Therefore, LCMV GP transcripts in these tg mice should a priori not differ from normal genomic transcripts and are clearly distinct from viral RNA transcripts generated during viral infection. Although IFN‐β mRNA levels were considerably increased (∼6‐fold) in spleen of MHCI.GP^+^ mice, it is important to emphasize that these levels were orders of magnitude lower compared to those in acutely LCMV‐infected mice [43]. In distinction to LCMV infection, MHCI.GP^+^ mice also did not exhibit increased IFN‐α transcript levels. Hence, the LCMV GP‐induced IFN‐I upregulation studied here is much more comparable to microbiota‐mediated homeostatic IFN‐I regulation [8] than to the IFN‐I response induced by acute LCMV infection.

LCMV is an enveloped virus, and for cell entry, LCMV GP expressed on the surface of viral particles binds to α‐dystroglycan, one of potentially many more cognate virus receptors for LCMV [44]. More recently, Axl, Tyro3, DC‐SIGN, and LSECtin were proposed to enhance LCMV GP‐mediated pseudotype infection [45]. It is, however, still a matter of debate whether these molecules act as real viral entry receptors or whether they mainly function to concentrate LCMV on the cell surface [46]. Importantly, none of these potential receptors for LCMV GP, including α‐dystroglycan, has been associated with IFN‐I induction in the literature.

Several viruses, including HCMV, MCMV, HSV, HCV, measles, vaccinia, but also LCMV, were reported to encode proteins capable of stimulating TLR2 [47]. Our analysis of TLR2‐deficient mice, however, showed that the LCMV GP‐induced upregulation of Sca‐1 in CD8 T cells and of CD44 and CXCR3 in CD4 T cells was TLR2‐independent. This excludes a role of this sensor in our model system. At present, the mechanism of how LCMV GP expression in tg mice upregulates IFN‐β expression is unknown. The finding that the activated CD4 T cell phenotype and the upregulated Sca‐1 levels in MHCI.GP^+^ mice were partially IFNAR1‐independent further suggests that tg LCMV GP expression induces additional factors with activation potential besides IFN‐β. IL‐27 may represent one of these factors because it is also capable of upregulating Sca‐1 levels in T cells [20].

In MHCI.GP^+^ mice, IFN‐β transcripts were upregulated in total splenic tissue, splenic CD11b^+^, and CD11c^+^ cells but not in pDCs despite their physiologically higher IFN‐β transcript levels. Various cell types, including fibroblasts, epithelial cells, macrophages, and DCs, are known to express IFN‐β [48]. Weak IFN‐I signals enhance the IFN‐I signaling pathway by sustaining the expression of the transcription factor IRF‐7 that is essential for efficient IFN‐I production after stimulation [49, 50, 51]. Interestingly, the RNA‐seq experiments revealed that IRF‐7 transcript levels were increased about 3.6‐fold in MHCI.GP^+^ mice. This indicates that the increased IFN‐β levels in these tg mice can initiate this amplification loop, possibly leading to a synergistic effect after stimulation with ligands of pattern‐recognition receptors.

In mice congenitally infected with LCMV, and therefore life‐long carriers of LCMV, IFN‐I is produced at low but elevated levels [52]. The triggers that sustain this chronic IFN‐I production in these LCMV carrier mice are unknown. Although LCMV GP is downregulated in LCMV carrier mice [53], systemic persistence of LCMV depends on continuous viral spread and reinfection [54]. Hence, it is possible that the LCMV GP‐induced IFN‐β production, as demonstrated here in tg mice, contributes to the observed enhanced IFN‐I production in persistently infected LCMV carrier mice. LCMV is a natural mouse pathogen, and significant portions of wild mice are congenitally infected with LCMV [55]. Our RNA‐seq analysis revealed an increase in several ISGs in MHCI.GP^+^ that are effective in the defense against viruses, bacteria, and parasites. Enhanced constitutive IFN‐I production and upregulated ISGs as modeled here in LCMV GP‐tg mice may therefore represent a selection advantage of persistently LCMV‐infected mice living in the wild. Indeed, a study published more than 60 years ago showed that LCMV carrier mice can exhibit heterologous viral interference [56].

LCMV GP‐expressing mice had more MP CD4 T cells and more effector regulatory CD4 T cells, and this activation phenotype was, at least partially, IFN‐I signaling dependent. How could upregulated IFN‐β levels induce this activated CD4 T cell phenotype? It is possible that enhanced direct IFN‐I signaling in self‐antigen‐driven CD4 T cell subsets enhances their survival. Marrack et al. [57] showed that in vitro activated T cells could be kept alive with IFN‐I by a mechanism that did not involve bcl‐2, bcl‐XL, or other cytokines. Direct IFN‐I signaling in CD4 T cells can also sustain clonal expansion after viral infections in vivo [58]. Besides providing the third signal for T cell activation, direct IFN‐I signaling may also protect antiviral CD4 T cells from NK cell‐mediated killing [59]. Remarkably, 2W peptide immunization induced 2W‐specific effector/memory CD4 T cells in MHCI.GP^+^ and tg‐negative littermates to the same extent. The survival‐enhancing effect of increased direct IFN‐I signaling in T cells may therefore primarily operate in MP and regulatory CD4 T cells that have high turnover rates due to self‐antigen‐driven homeostatic proliferation.

In addition to direct IFN‐I signaling effects in CD4 T cells, other IFN‐I‐dependent mechanisms may also play a role. The RNA‐seq experiments revealed that up to ∼80 ISGs potentially coding for self‐antigens were upregulated at least two‐fold at transcriptional levels in MHC.GP^+^ mice. Moreover, three of the five most upregulated genes (*Ifi44, Gm4841*, and *lipgp1c*) were not or only very slightly induced in the thymi of MHC.GP^+^ mice. In MHCI.GP^+^ mice, low affinity T cells specific for these self‐antigens may escape thymic negative selection but will be confronted in the periphery with high levels of them that could lead to their activation and homeostatic expansion. In wt mice, such low affinity T cells will also pass thymic negative selection, but will not meet high levels of the corresponding self‐antigens in the periphery. In addition, MHC‐II expression in B cells of MHCI.GP^+^ mice was increased by ∼30% (mean fluorescence intensity [MFI] after antibody staining), most likely due to increased IFN‐β levels. This could further promote self‐antigen‐driven homeostatic proliferation of CD4 T cells. Finally, IFN‐I signaling in DCs may exert enhancing effects in differentiation and antigen presentation [60]. Taken together, enhanced self‐antigen presentation may also represent an important factor for the activated CD4 T cell phenotype in LCMV GP‐tg mice. The finding that the TCR Vβ repertoire representation of CD44^high^CD4 T cells in MHCI.GP^+^ mice is distinct from tg‐negative littermates, providing further support for this interpretation.

It is noteworthy that MHC‐I expression in B cells of MHCI.GP^+^ mice was also increased by ∼80% (MFI after antibody staining), but CD8 T cells in these mice did not exhibit a more activated phenotype besides upregulated Sca‐1 and Ly6C expression. As demonstrated previously in Nur77‐GFP reporter mice, CD4 T cells inherently show stronger TCR signals than CD8 T cells in the steady state [61]. It is therefore possible that an increased self‐antigen presentation in MHCI.GP^+^ mice is only sufficient to alter CD4 but not CD8 T cell homeostasis.

## 4 Data Limitations and Perspectives

Our data demonstrate that expression of LCMV GP in vivo increases constitutive IFN‐β levels and upregulates numerous ISGs, but the pathway recognizing LCMV GP and inducing these effects is not yet identified. Furthermore, it is unknown whether expression of other viral or microbial genes exert similar effects as LCMV GP. To address these questions, cell culture systems capable of mimicking LCMV GP‐induced IFN‐β upregulation would be very useful. Our data further reveal that the increased constitutive IFN‐β levels were, at least partially, responsible for the altered CD4 T cell homeostasis. Yet, it remains open whether direct IFN‐I signaling in T cells and/or enhanced self‐antigen presentation played the key roles for these alterations. Finally, it will be interesting to examine whether the upregulated ISG levels in LCMV GP‐tg mice and possibly also in LCMV carrier mice are associated with an increased resistance to pathogen infections or tumor challenge.

## 5 Materials and Methods

### 5.1 Mice

C57BL/6J (B6) and 129S2/SVPasCrl (129S2/Sv) mice were maintained at the Max Planck Institute of Immunobiology and Epigenetics. MHCI.GP (GP_WE_‐tg, [62]) and CAG.GP [63] mice were described previously. B.GP and DC.GP mice were generated by mating Stop‐GP^flox^ [23] with mb1‐cre [24] and CD11c‐cre [25] mice, respectively. MHCI.GP;TLR2^−/−^ and MHCI.GP;Ifnar1^−/−^ mice were generated by breeding with TLR2^−/−^ [64] and Ifnar1^−/−^ [65] mice, respectively. Mice were typed by PCR using the following primers: MHCI.GP: 5′‐CGACGGCAAGACCACCTGGTGC and 5′‐GTTCACGGTGGTCTTGAACACGTGC; CAG.GP: 5′‐GGCGCCGGCAGGAAGGAAAT and 5′‐CCGTACATGCCACAGGACCTACC; mb1‐cre: 5′‐ACCTCTGATGAAGTCAGGAAG AAC and 5′‐GGAGATGTCCTTCACTCTGATTCT; CD11c‐cre: 5′‐ACTTGGCAGCTGTCTCCAAG and 5′‐GCGAACATCTTCAGGTTCTG; TLR2^−/−^: 5′‐CTTCCTGAATTTGTCCAGTACA, 5′‐GGGCCA GCTCATTCCTCCCAC and 5′‐ACGAGCAAGATCAACAGGAGA; Ifnar1^−/−^: 5′‐CGAGGCGAAGTGGTTAAAAG, 5′‐ ACGGATCAACCTCATTCCAC and 5′‐AATTCGCCAATGACAAGACG. PCR conditions: 95°C for 3 min; 35 cycles of 95°C for 15 s, 60°C for 15 s, 72°C for 15 s; and final extension 72°C for 1 min using KAPA Mouse Genotyping Kit (KAPABIOSYTEMS). For adoptive transfer, 5 × 10^7^ total spleen cells from B6.Thy1.1^+^ mice were injected intraperitoneally into recipient mice. To block IFN signaling, recipient mice were treated twice (day‐1 and day+2 after cell transfer) with 1 mg anti‐IFNAR monoclonal antibody (mAb) (clone MAR1‐5A3, Leinco Technologies). For peptide immunization, mice were injected intraperitoneally with 20 µg 2W peptide (EAWGALANWAVDSA, Genaxxon) together with 20 µg Poly (I:C) (Miltenyi). All mouse lines used here were kept on a B6 background unless stated otherwise. All mice were housed under specific pathogen‐free conditions and used for analysis at 7–30 weeks of age.

### 5.2 Flow Cytometry

Spleen cells (10^5^–10^6^ in 50–100 µL) were stained with appropriately diluted fluorescently labeled mAb (0.1–1 µg in 50–100 µL) in PBS containing 2% FCS and 0.1% NaN_3_ at 4°C for 30 min. mAb specific for Bcl‐2‐AF647 (BCI/10C4), CD8α‐FITC (53‐6.7), CD8α‐PE‐Cy7 (53‐6.7), CD44‐BV785 (IM7), CD44‐PE (IM7), CD45R/B220‐BV421 (RA3‐6B2), CD62L‐BV510 (MEL‐14), CD86‐PE (GL‐1), CD90.1/Thy1.1‐BV650 (OX‐7), CD95‐APC (SA367H8), CD103‐BV785 (2E7), CXCR3‐PE (CXCR3‐173), CXCR3‐BV421 (CXCR3‐173), GL7‐PE (GL7), ICOS‐PE/Cy7 (7E.17G9), IgD‐APC (11‐26c2a), IgM‐FITC (RMM‐1), Ki‐67‐AF647 (16A8), Ly6A/E (Sca‐1)‐AF647 (D7), MHCI (K^b^)‐APC (AF6‐88.5), PD‐1‐BV785 (29F.1A12), PD‐1‐PE‐Fire810 (29F.1A12), Vβ2‐PE (B20.6), Vβ5.1/5.2‐APC (MR9‐4), Vβ8.1/8.2‐FITC (KJ16‐133.18), Vβ11‐AF647 (KT11), and Vβ12‐PE (MR11‐1) were purchased from Biolegend; mAb specific for CD8α‐PE (53‐6.7), Foxp3‐BV421 (FJK‐16s), and MHCII (I‐A^b^)‐PE (AF6‐120) from Thermofisher; mAb specific for CD4‐BV650 (RMA4‐5), Foxp3‐R718 (3G3), and ICOS‐BUV805 (C398.4A) from BD Bioscience. The LCMV GP‐specific mAb KL25 [66] was purified from hybridoma supernatant and labeled with AF647 in house. PE‐labeled I‐A^b^ tetramers complexed with gp66 (DIYKGVYQFKSV) and 2W (EAWGALANWAVDSA) peptides were obtained from the NIH Tetramer Facility (Emory). Spleen cells were stained with tetramers at room temperature for 1 h. gp66:I‐A^b^‐tetramer‐stained cells from naïve mice were enriched on magnetized columns (Miltenyi) with magnetic beads conjugated with anti‐PE antibodies as described [63, 67]. Samples were measured on an LSRFortessa or FACSymphony A5 SE flow cytometer (BD Biosciences) and analyzed by FlowJo software 887 (Tree Star). Flow cytometric analysis was performed in accordance with the guidelines for the use of flow cytometry and cell sorting in immunological studies [68] and gating strategies are shown in Figure S8.

### 5.3 Quantitative RT‐PCR

Total RNA was extracted from entire organs (spleen/thymus) and from isolated splenic CD8^+^, CD11b^+^ and CD11c^+^ cells with TRI reagent (Sigma‐Aldrich), treated with TURBO DNase (Thermo Fisher), and reverse‐transcribed with SuperScript IV Reverse Transcriptase (Thermo Fisher) following the instructions of the manufacturers. CD8^+^, CD11b^+^, and CD11c^+^ cells from spleen were isolated using positive selection with microbeads and magnetized columns (Miltenyi). pDCs were isolated using negative selection with pDC Isolation Kit (Miltenyi), and total RNA was isolated with RNeasy Plus Micro Kit (QIAGEN). PCR amplification was performed in a 7500/7500 Fast Real‐Time PCR machine (Applied Biosystems) with Absolute Blue qPCR SYBR low ROX mix (Thermo Fisher). For IFN quantification, the following primer pairs were used: IFNα (pan): 5′‐CCACAGGATCACTGTGTACCTGAGA and 5′‐CTGATCACCTCCCAGGCACAG; IFNβ: 5′‐GTACGTCTCCTGGATGAACTCC and 5′‐CCACGTCAATCTTTCCTCTTGC; HPRT: 5′‐TGGATACAGGCCAGACTTTGTT and 5′‐CAGATTCAACTTGCGCTCATC. For ISG quantification, the following primer pairs were used: Echdc2: 5′‐GGGCTAATTGAGACCACTCG and 5′‐GATTTACCAGGCCCAACTCA; Gm4841: 5′‐TCTGTGCATCCTGCTCTGAGTG and 5′‐GGTGTGTGTGTGTGTGTGTGTG; Ifi44: 5′‐AACTGACTGCTCGCAATAATGT and 5′‐GTAACACAGCAATGCCTCTTGT; Iigp1c: 5′‐GTAGACTCAAGCCAAGAGCA and 5′‐AGCAGAAACTTTAAACAGTGGG; Zyg11b: 5′‐CTGGGATGAGAAACCACCCT and 5′‐TTGAGCAGCAAATGGGTCAC. Values were normalized to HPRT.

### 5.4 Bulk RNA‐Sequencing

Total RNA was extracted from splenic lymphocytes (CD4^+^, CD8^+^, and CD19^+^ cells) of MHCI.GP^+^ and MHCI.GP^−^ mice with TRI reagent (Sigma‐Aldrich). Sample quality measurements and library preparation were carried out by the Deep Sequencing facility at Max Planck Institute of Immunobiology and Epigenetics, Freiburg. Libraries were prepared using the Illumina Stranded mRNA Prep Kit (Ligation) and sequenced to a depth of 20–30 million paired end (2 × 100 bp) reads on a NovaSeq 6000 instrument. Adapters were trimmed using cutadapt v4.9 [69]. Trimmed reads were aligned to the primary GRCm39 assembly from Ensembl using STAR 2.7.11b [70]. Gene annotations were fetched from Ensembl (version 112). Annotations of repeat elements were generated using RepeatMasker v4.1.5 (Smit Arian F. A., Hubley Robert, and Green Philip. RepeatMasker Home Page, 2013. http://www.repeatmasker.org/). Output from RepeatMasker was further processed, and suffixes such as “‐LTR” and “‐int” were stripped to arrive at matching names between LTRs and internal parts of LTR/ERV elements. The resulting RepeatMasker GTF file was further filtered to retain elements of class DNA, SINE, LINE, or LTR/ERV and concatenated with the Ensembl GTF. Mapped reads were counted using featureCounts (subread 2.0.6 [71], allowing multi‐mapping reads and allocating fractional counts (‐p–countReadPairs ‐B ‐O ‐M –fraction). Counts were summarized to the gene level. For RepeatMasker elements, summarization was performed on the basis of element names. Genes were kept in the count matrix if they had a count of at least 1 in at least half of all samples. Differential expression analysis was performed with DESeq2 1.42.0 [72]. Genes with a confident |log_2_(fold change)| ≥ 1 were identified by moderation of log_2_ fold changes with ashr 2.2.63 (lfcThreshold = 1) [73]. Genes were deemed significantly differentially expressed with an *s* value ≤0.01. ORA was performed with fgsea 1.28.0 [74] with GO gene sets from MSigDB M5 [75] or the RepeatMasker classes DNA, SINE, LINE, and LTR/ERV. All genes, available in the data set, were considered for the background gene universe. Heatmaps were generated with ComplexHeatmap 2.18.0 [76]. Code to reproduce the analysis was deposited at https://www.github.com/osthomas/rnaseq_gp.

### 5.5 Statistical Analysis

Other statistical analysis was carried out using GraphPad Prism (version 8.3.1). The tests used are indicated in the respective figure legends.

## Author Contributions

Hanspeter Pircher designed research, performed experiments, analyzed data, and wrote the article; Oliver S. Thomas analyzed data and wrote the article; Anne S. Haefke performed experiments and analyzed data; Annette Oxenius analyzed data and edited the manuscript; Thomas Boehm analyzed data and edited the manuscript.

## Ethics Statement

The animal experiments were approved by the Regierungspräsidium Freiburg (licenses 35‐9185.81; G‐15/15, G‐19/175, G‐20/56, G‐22/010) and performed in accordance with the German animal protection law and the directive 2010/63/EU of the European Parliament and the by the Cantonal Veterinary Office of Zurich, Switzerland (ZH097/23).

## Conflicts of Interest

The authors declare no conflicts of interest.

## Supporting information




**Supporting File 1**: eji70188‐sup‐0001‐SupMat.pdf.

**Supporting File 2**: eji70188‐sup‐0002‐tableS1.xlsx.

**Supporting File 3**: eji70188‐sup‐0003‐tableS2.xlsx.

## Data Availability

RNAseq data were deposited on GEO (accession number GSE303494). Results of differential expression and overrepresentation analyses are included as Supporting Information.
