# Supplementary material for: Increased Constitutive Interferon‐β Levels and Altered CD4 T Cell Homeostasis Induced by Expression of a Viral Glycoprotein
Source: Eur J Immunol. 2026 Apr 13;56:e70188. doi: 10.1002/eji.70188 (PMC13073064; doi:10.1002/eji.70188)
Supplement: Supplementary file 1 — Supporting File 1: eji70188‐sup‐0001‐SupMat.pdf. [file EJI-56-e70188-s001.pdf]

Supporting Information

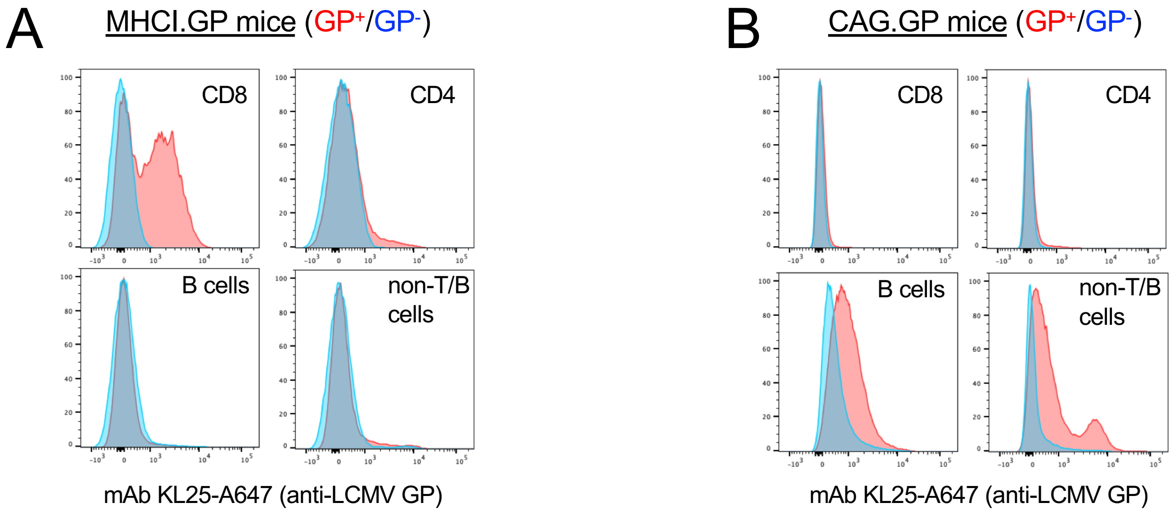

Figure S1. LCMV GP expression in LCMV GP tg-mice. Expression was determined by cell surface staining with the LCMV GP-specific monoclonal antibody (mAb) KL25. Shown are representative flow cytometry histograms gated on the indicated splenic cell subsets from MHCI.GP$^+$ (red) and MHCI.GP$^-$ (blue) (A) and from CAG.GP$^+$ (red) and CAG.GP$^-$ (blue) (B) mice.

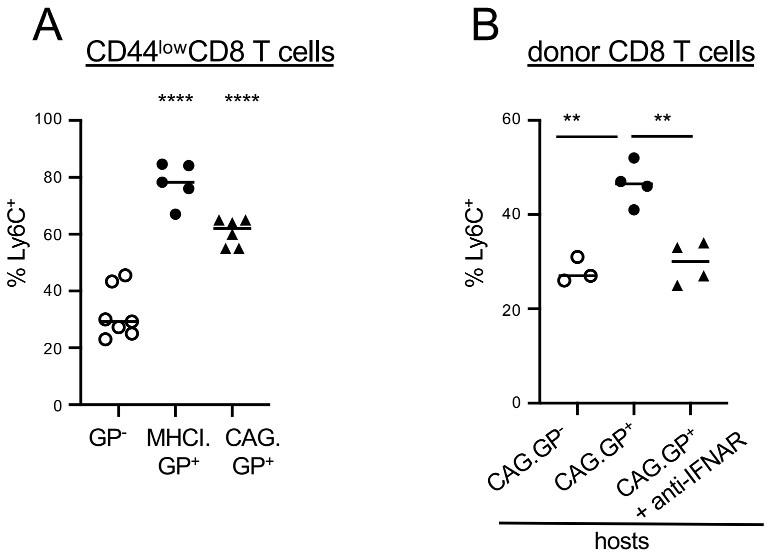

Figure S2. Ly6C expression in splenic CD8 T cells from LCMV GP-tg mice. (A) Percentages of $Ly6C^+$ cells of naive-phenotype $CD44^{low}CD8$ T cells from $MHCI.GP^+$ (filled circles), $CAG.GP^+$ (filled triangles) and GP^- littermate control mice (open circles). (B) Percentages of $Ly6C^+$ cells of donor CD8 T cells from wild type $B6.Thy1.1^+$ mice after adoptive transfer (day 5) into host mice with the indicated genotypes. Values from individual mice are displayed. Data are pooled from 2-4 independent experiments for each group (n=3-7 per group). Statistical tests: **$p < 0.01$; ****$p < 0.0001$; unpaired t-test.

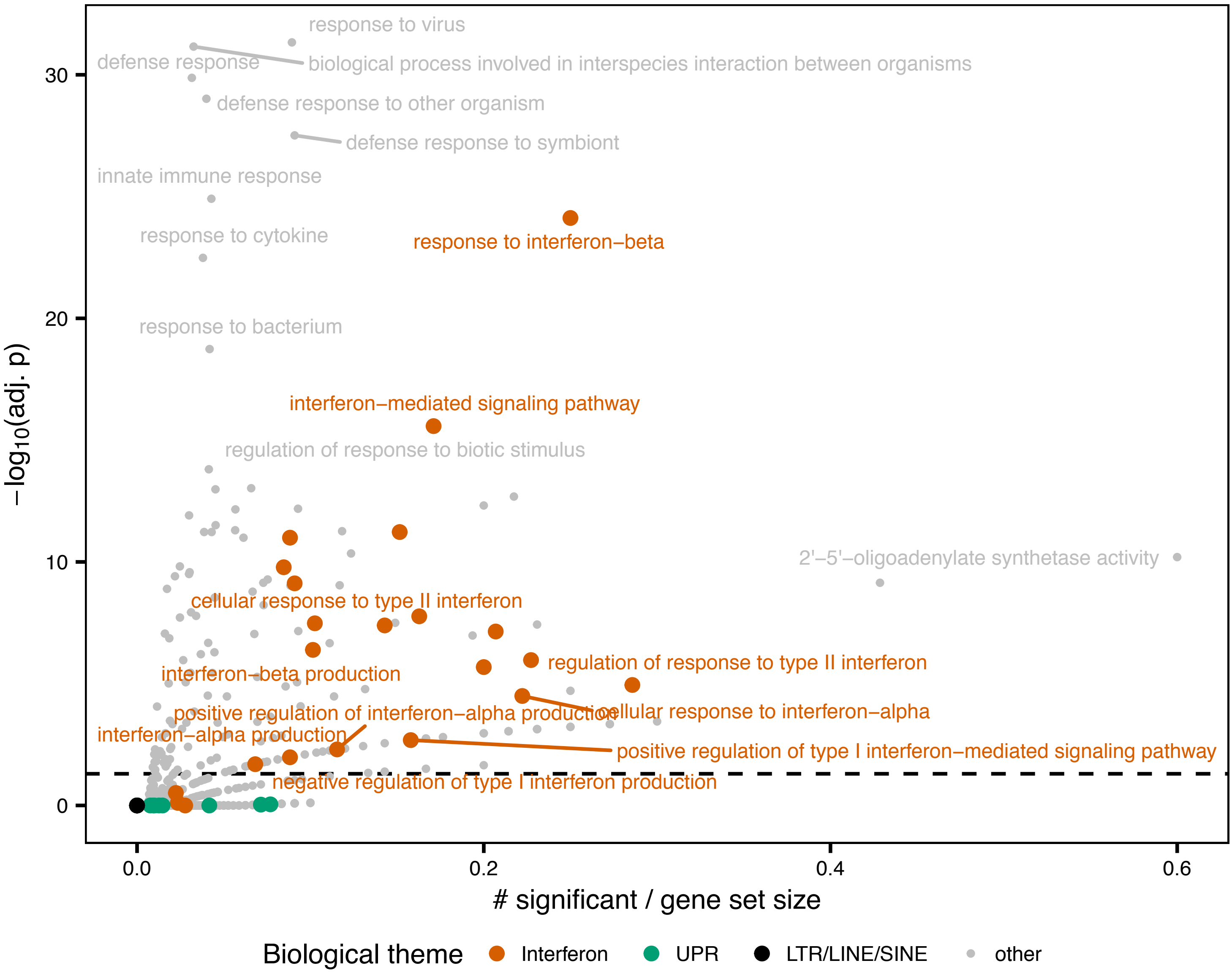

Figure S3. Results of overrepresentation (ORA) analysis of significant genes in Gene Ontology gene sets (MSigDB M5) and RepeatMasker classes DNA, LINE, SINE, LTR/ERV, showing fraction of significant genes by -$\log_{10}$(adj. p) per set. Dashed line at $p = 0.05$. Biological themes related to interferon (red; gene set name contains „IFN“ or „Interferon“), unfolded protein response (UPR, green; offspring of GO term „response to topologically incorrect protein“) or LINEs/SINEs/ERVs (black; from RepeatMasker) are indicated.

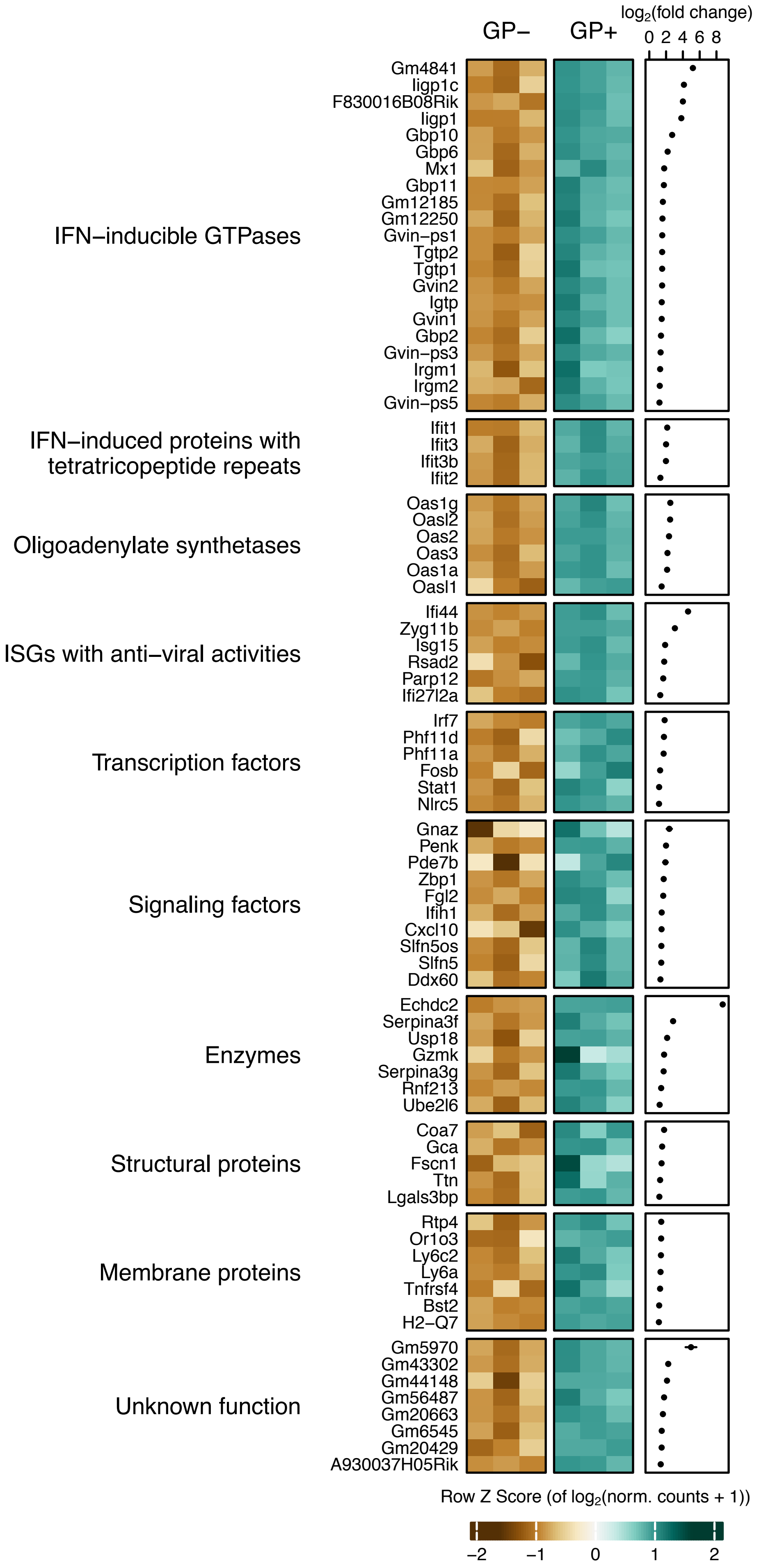

Figure S4. Heatmap showing the Row Z score of log2 normalized counts (DESeq2 normalization) of differentially upregulated genes (s-value ≤ 0.01 for a log2(fold change) ≥ 1) in splenic lymphocytes from MHCI.GP^{+} vs. MHCI.GP^{-} mice.

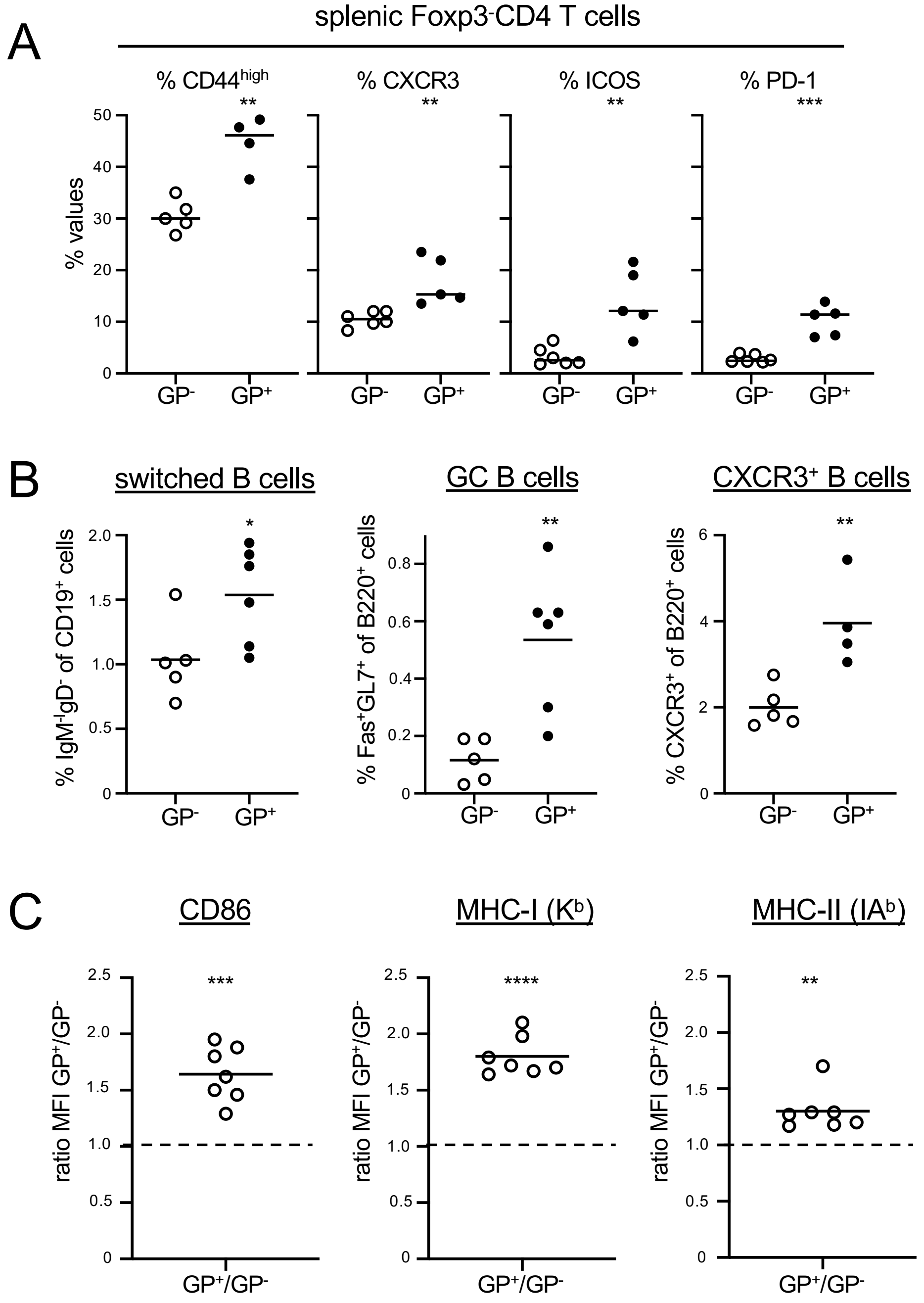

Figure S5. (A) Expression of the indicated markers in percent of splenic Foxp3-CD4 T cells from MHCI.GP+ (filled circles) and MHCI.GP- (open circles) mice. (B) Percentages of switched memory (IgM-IgD-), of germinal center (Fas+GL7+) and of CXCR3+ cells of splenic B cells from MHCI.GP+ (filled circles) and MHCI.GP- (open circles) mice. Values from individual mice are displayed. Data are pooled from 4 independent experiments for each group (n=4-6 per group). (C) Mean fluorescence intensity (MFI) ratios of CD86, MHC-I (K^b) and MHC-II (I-A^b) expression in B cells. Shown are MFI ratios calculated on pairs of B220+ B cells from MHCI.GP+ and MHCI.GP- mice that were analyzed in parallel. Statistical tests: unpaired t-test for (A, B) and single sample t-test for (C) for the significance between observed means and the hypothetical mean of 1. *$p < 0.05$; **$p < 0.01$; ***$p < 0.001$; ****$p < 0.0001$.

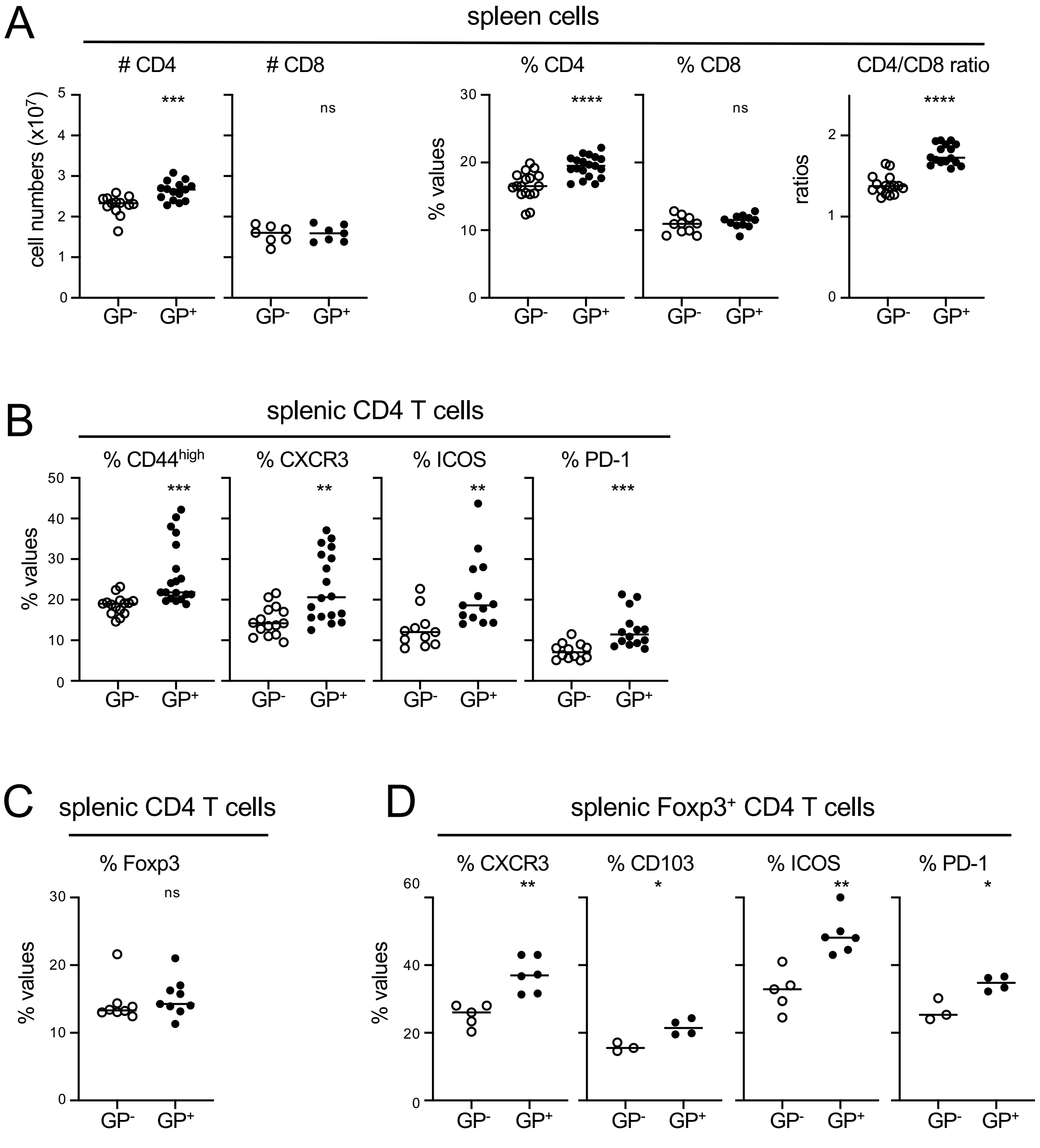

Figure S6. Increase of CD4 T cells with an activated phenotype in CAG.GP$^+$ mice. (A) Absolute cell numbers and percentages of the indicated cell subsets in spleen of CAG.GP$^+$ (filled circles) and CAG.GP$^-$ (open circles) littermate mice. (B) Expression of the indicated markers in percent of splenic CD4 T cells. (C) Percentages Foxp3$^+$ cells of splenic CD4 T cells. (D) Expression of the indicated markers in percent of splenic Foxp3$^+$CD4 T cells. Values from individual mice are displayed. Data are pooled from 3-12 independent experiments for each group (n=3-20 per group). Statistical tests: ns, not significant; *p < 0.05; **p < 0.01; ***p < 0.001; ****p < 0.0001; unpaired t-test.

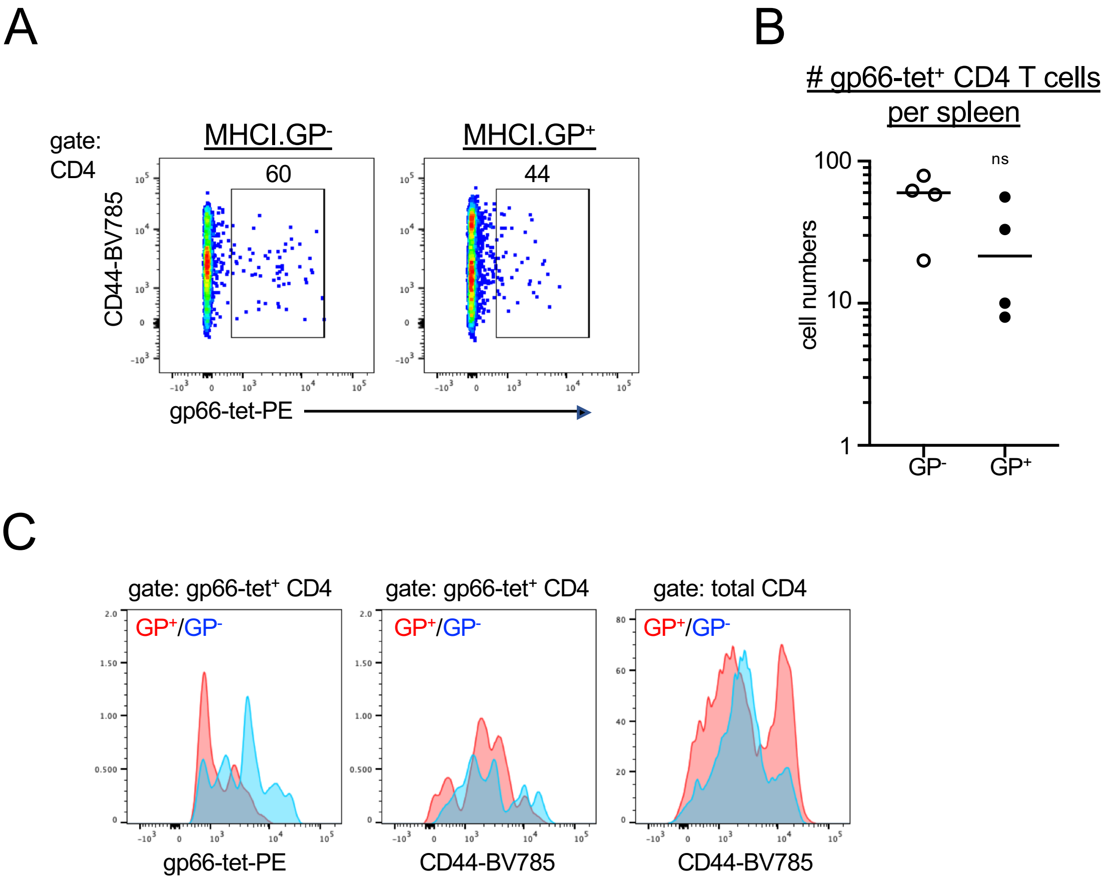

Figure S7. Analysis of LCMV GP-specific CD4 T cells in MHCI.GP$^+$ and MHCI.GP$^-$ mice. (A) Representative flow cytometry dot plots gated on splenic CD4 T cells following gp66/I-A^b tet-based enrichment. The numbers above the gate indicate total numbers of gp66-tet$^+$ cells recovered from the spleen of one mouse in the sample shown. (B) Total numbers of gp66-tet$^+$ CD4 spleen cells isolated from spleen of mice with the indicated genotype. Values from individual mice are displayed. (C) Flow cytometry histograms from (A) gated on the indicated splenic cell subsets from MHCI.GP$^+$ (red) and MHCI.GP$^-$ (blue) mice after gp66/I-A^b tet-based enrichment. Data are pooled from 4 independent experiments for each group (n=4 per group). Statistical tests: ns, not significant; unpaired t-test.

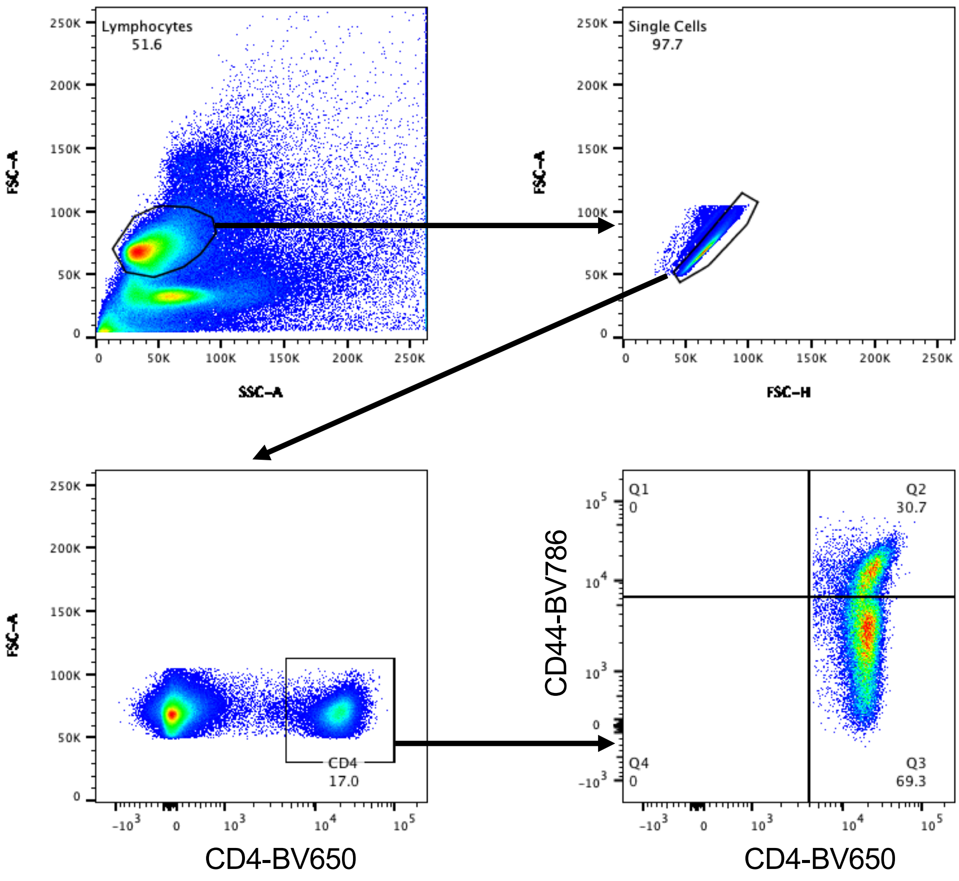

Figure S8. Gating strategy to determine expression of activation markers in CD4 T cells.
